# A Retrospective Cross-Sectional Analysis of Abnormal Cervical Mechanics in Patients With Scoliosis

**DOI:** 10.7759/cureus.91098

**Published:** 2025-08-27

**Authors:** Justin M Dick

**Affiliations:** 1 Orthopedics, Clear Life Scoliosis Reduction and Chiropractic, Charlotte, USA

**Keywords:** abnormal x-ray, adolescent idiopathic scoliosis (ais), cervical assessment, spine curvature disorders, etiology

## Abstract

Introduction: Adolescent Idiopathic Scoliosis (AIS) is a complex spinal deformity that affects millions around the globe. Prevailing theories limit the scope of the spinal deformity to only the scoliotic affected area.

Objective: The aim of this study is to investigate the biomechanical relationship between cervical mechanics and AIS. Research is warranted to evaluate the specific mechanical correlations and potential contributory role of altered dynamic cervical alignment in the development or progression of scoliotic deformities.

Methods: This study was designed as a cross-sectional, descriptive, and retrospective analysis. This study included 37 participants for analysis. Cervical Lateral (Neutral, Flexion, Extension), AP scoliosis radiographs with computer-aided quantitative measurements were recorded for cervical lordosis, cervical segmental instability, and scoliosis Cobb angles. Anterior head translation (AHT) was measured from C2 to C7. Global cervical shape was compared to known buckling patterning. Quantification of the scoliosis utilized the Cobb scoliosis method of measurement.

Results: This study's data strongly support the hypothesis that all AIS patients exhibit a loss of cervical lordosis, with cervical instability contributing to abnormal biomechanics associated with cervical buckling. A high percentage of the participants measured abnormal segmental translation, highest at 70.3% C3-C4 (p=0.0011). A lower proportion of patients (21.6%, p=0.9999) met the threshold for segmental angulation. The majority of patients (33 out of 37; 89.2%) exhibited Order 1 buckling. Four patients (10.8%) demonstrated Order 2 buckling.

Conclusions: This case series demonstrates a correlation between AIS participants, cervical instability, cervical hypolordosis, and cervical buckling. This study adds to the literature by reviewing the abnormal cervical mechanics in AIS patients.

## Introduction

Adolescent idiopathic scoliosis (AIS), found between 11 and 18 years of age, is one of the most common spinal deformities but has an unknown cause, affecting 2%-3% of the general population [1]. The female-to-male ratio ranges from 1.5:1 to 3:1 and rises significantly with age [2]. An estimated 20% of scoliosis cases are attributable to identifiable etiologies such as neuromuscular, syndromic, or congenital anomalies, whereas approximately 80% are classified as idiopathic scoliosis. AIS is defined on radiographs as a three-dimensional spinal deformity measured by a Cobb angle greater than 10 degrees with vertebral rotation and coronal malposition [2].

Scoliosis is known to impact the quality of one's life and carries financial burdens associated with medical costs. In the Netherlands, the annual societal cost per AIS patient was equivalent to $13332, with the highest expenses attributed to healthcare and productivity losses. Among adult participants, 10% experienced severe pain, while 44% reported moderate pain or discomfort. Statistically significant differences in sector costs and health-related quality of life (HRQoL) were observed across age groups. The utility score ranges from 0, indicating death, to 1.0, indicating perfect health. The average utility score for adults was 0.7 (SD 0.20) [3]. As scoliosis progresses, disability increases, and in more severe cases, it may lead to thoracic insufficiency syndrome (TIS) [4,5]. 

The National Institutes of Health (NIH) identifies several physical indicators of scoliosis, including uneven shoulder height, a more prominent or protruding shoulder blade on one side, an asymmetrically raised hip, and an uneven rib cage appearance when bending forward [6]. These signs can indicate spinal curvature and warrant further advanced imaging techniques. Evidence has shown that spinal deformities are found in correlation with other spinal deformities. Studies have shown a higher incidence of cervical hypolordosis and/or kyphosis in people with AIS, 89% [7]. Cervical structural abnormalities that may result in clinical symptomatology include, but are not limited to, coronal (e.g., scoliosis) and sagittal plane deformities such as kyphosis, hyperlordosis, spondylolisthesis, and retrolisthesis [8]. Cervical spine deformity is associated with substantial clinical morbidity, including heightened pain levels, restricted mobility and functional capacity, and diminished ability to engage in occupational and activities of daily living [9-11].

Cervical instability is the failure of the cervical spine to maintain its normal pattern of intervertebral alignment under physiological loads [12]. Disruption of cervical alignment with instability initiates compensatory adaptations along the spinal kinetic chain, with alterations typically progressing caudally from the cervical region [12]. The instability then causes structural integrity to be compromised, resulting in impaired posture, neck pain, dizziness, aural symptoms, and increased vulnerability of neurovascular and spinal structures to physiological loads [13,14]. Persistent instability may lead to progressive osseous and soft tissue changes, nerve root or spinal cord involvement, and a decline in neuromuscular function and quality of life [15]. These contribute to the economic burden encompassing direct medical costs, decreased workplace productivity, and employment-related challenges. In 2016, low back and neck pain represented the highest health care expenditure among the 154 evaluated medical conditions in the United States [16]. In 2012, neck pain contributed to work absences in approximately 25.5 million Americans, resulting in a mean of 11.4 lost workdays per affected individual annually [17]. 

However, cervical instability in people with AIS is an understudied topic. This investigation seeks to characterize the frequency and morphological presentation of cervical spine instability and buckling in a cohort of AIS patients. This correlation can help further develop treatment methods and a full spine scoliosis analysis. 

## Materials and methods

Institutional Review Board (IRB) approval was not required due to the retrospective design of this study, consistent with the exemption criteria outlined in the common rule (45 CFR 46.104). Standard of care informed consent was obtained from the patient prior to publication. The report contains no identifiable personal information; therefore, the Declaration of Helsinki does not apply to this case report. This retrospective analysis was conducted at a private healthcare facility in Huntersville, NC, utilizing radiographic data collected from radiographs (2021-2024).

Radiographic measurements were collected for cervical translation (C2-C7) and measured angulation (C2-C7). Descriptive statistics (mean, standard deviation, median, and range) were calculated for each parameter. The total translation and measured angle were derived through the maximum of the absolute value of C2-C7. Cervical buckling is defined as a force on the spine leading to abnormal biomechanics. This was further explained mathematically by Leonhard Euler and can be broken further into categories [18]. Clinical instability of the cervical spine (CICS) refers to the spine’s inability to maintain normal alignment and motion under physiological loads, resulting in the potential for neurological compromise, structural deformity, or significant pain [19].

To determine whether the observed cervical instability exceeded predefined abnormal thresholds, one-sample t-tests were conducted at a 95% confidence interval (p<0.05 considered statistically significant). All statistical analyses were performed using SAS 9.4 (SAS Institute Inc., Cary, NC, USA). Box plots were generated to visually depict the distribution of total translation and measured angles from C2 through C7.

Patient inclusion and exclusion criteria

This study included 37 radiographs taken from patients between 12 and 20 years of age. The following findings were consistently observed at the patients' initial visits. This design was to limit spinal hypermobility associated with adolescence and rigidity with the progression of age. Radiographs with a diagnosis of AIS with Cobb angle(s) greater than 10 degrees were included. All participants must have cervical lateral neutral, flexion, and extension views, as well as AP thoracic and lumbar radiographs. Radiographs demonstrated a Risser 2 or above. Structural spinal pathologies (e.g., wedged vertebrae, Ankylosing spondylitis (AS), and DISH) were not included in the study. Reported connective tissue disorders associated with hypermobility (e.g., Ehlers-Danlos syndrome (EDS), Marfan syndrome, and cerebral palsy (CP)) were also not included in this study. In this case series, radiographs were excluded if they presented with a history of cervical trauma, including motor vehicle collision, concussion, cervical acceleration-deceleration (CAD) syndrome (whiplash), or if the patient presented with a history of spinal surgical intervention.

Radiographs taken in this study were justified based on symptoms and subsequent physical examination. Several of the subjects were included based on incidental findings and did not include full spine X-rays to allow for Lenke classification. It is the expert opinion of the authors that cervical spine radiographs, including dynamic flexion and extension views, are crucial for AIS despite radiation concerns, as they reveal underlying dynamic mechanics (e.g., abnormal segmental translation and angulation) not visible on neutral views. Identifying the abnormal mechanics is vital as they can lead to significant pain, functional limitations, and may contraindicate certain physical activities, directly impacting patient prognosis and the efficacy of management strategies. Therefore, these detailed images are essential for a comprehensive biomechanical assessment of the entire spinal kinetic chain in AIS [20].

The validity and reproducibility of PostureRay® (PostureCo®, Inc., Trinity, Florida, USA) for radiographic measurements, including Cobb angle, translation, and angulation, are supported by its extensive use and validation in numerous published studies by Deed Harrison et al. and his research group, which consistently demonstrate its accuracy and reliability in quantitative spinal analysis [21,22].

**Figure 1:**
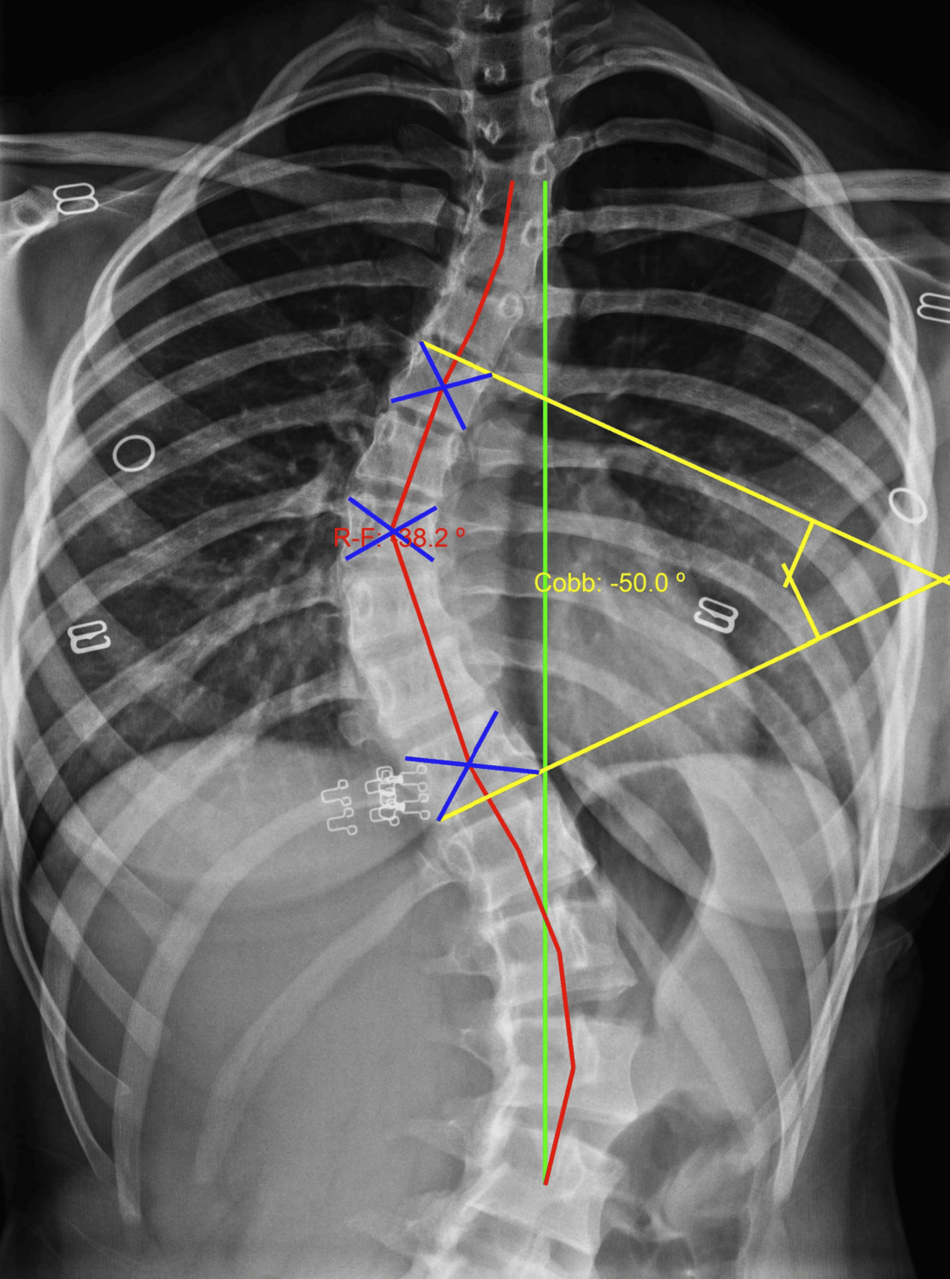
An AP thoracic/lumbar radiograph was taken at an FFD of 40 inches. The green line represents the normal spinal position, while the blue line indicates the center of the cross-section of the vertebrae. The red line represents the patient’s alignment, and the blue lines represent the projected centers of mass of the spine R-F: Risser-Ferguson method of analysis. Cobb: Cobb method of analysis with an apex at T8, a lower end vertebra at T11, and an upper measured vertebra at T6. FFD: focal film distance

Scoliosis is defined by a Cobb angle greater than 10°, measured on standard AP radiographs with PostureRay® (Figure 1) [2].

**Figure 2:**
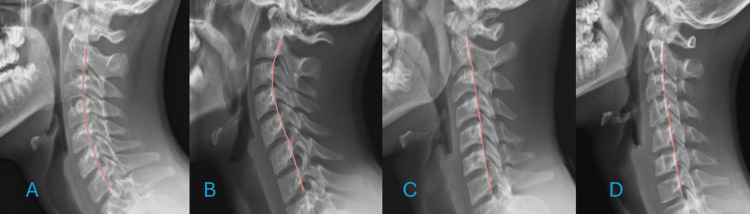
(A) Normal neutral cervical lordosis. (B) A first-order buckled mode is demonstrated, with flexion in the lower cervical spine and extension in the upper cervical spine. (C) An opposite first-order buckled mode, in which extension occurs in the lower cervical spine and flexion occurs in the upper cervical spine. (D) A second-order buckled mode, featuring extension in the lower cervical spine, flexion in the mid-cervical region, and extension in the upper cervical spine

Cervical buckling was classified into the following categories (Figure 2). This phenomenon can be further described using hierarchical buckling patterns, referred to as "orders."

Cervical instability

**Figure 3:**
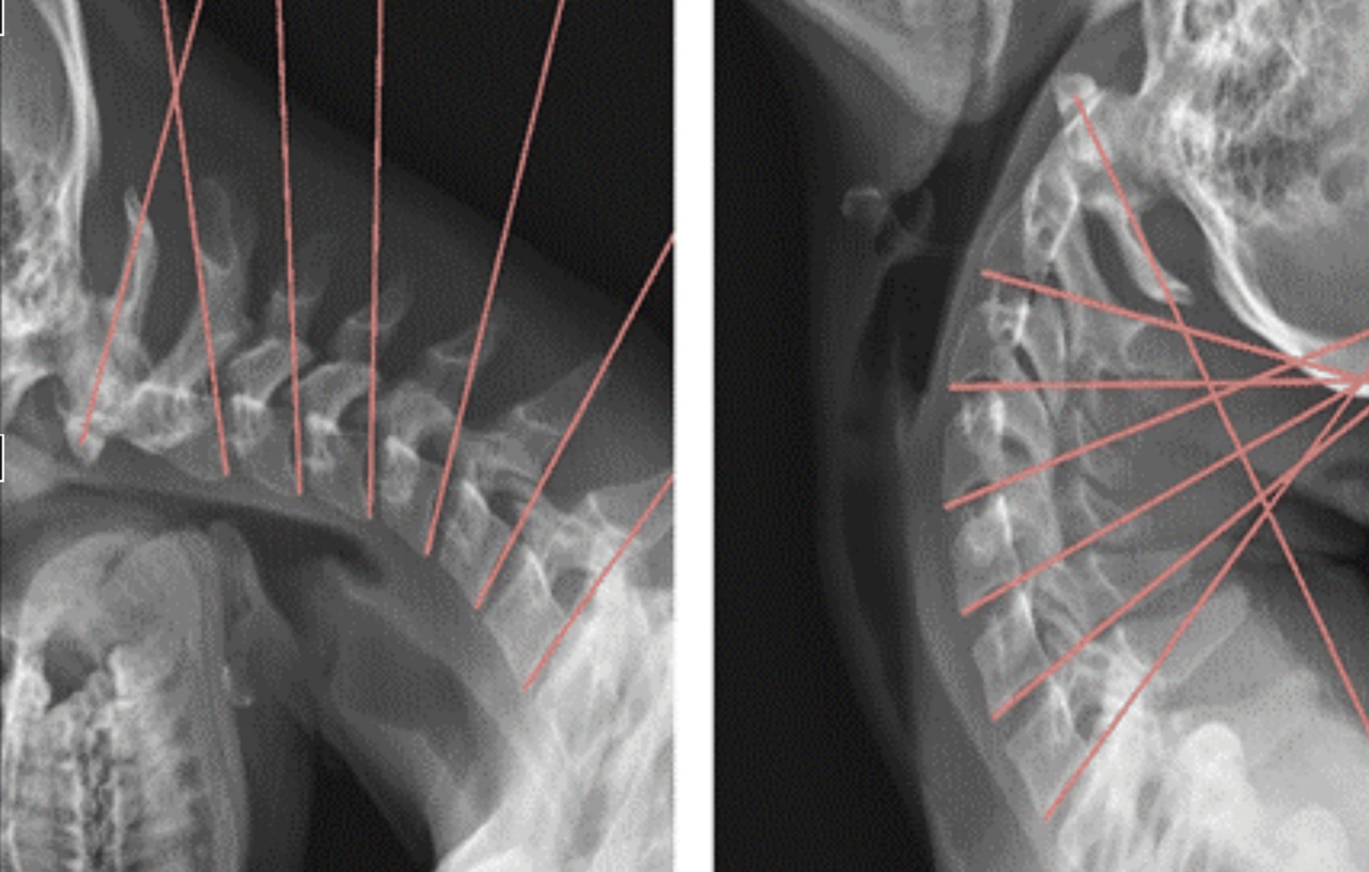
A motion segment of the spine is defined as two adjacent vertebrae, the intercalated disk, and the vertebral facet joint. Angular loss of integrity is defined as a difference in angular motion between two adjacent motion segments

**Figure 4:**
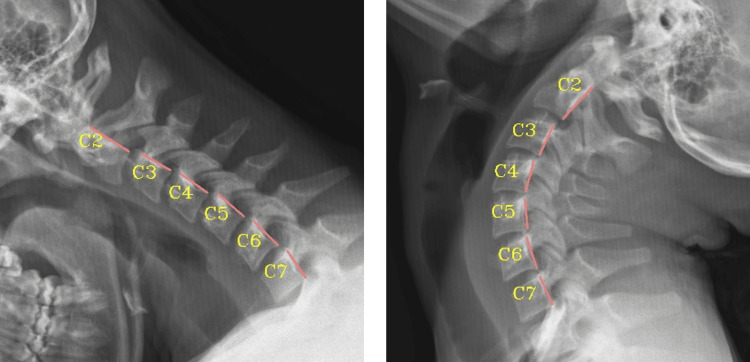
Translational motion is measured by determining the anteroposterior motion of one vertebra over another

An independent review of the radiographs was performed by medical radiologists through Spinal Kinetics (Spinal Kinetics LLC, Clearwater, Florida, US), utilizing Computerized Radiographic Mensuration Analysis (CRMA™). Angulation between adjacent vertebrae was considered abnormal when the following thresholds were reached: C2-3 >10°, C3-4 >14°, C4-5 >16.40°, C5-6 >14.90°, C6-7 >11.90° (Figure 3) [23]. Translation between adjacent vertebrae was considered abnormal when greater than the thresholds as follows: C2-3 >2.2 mm, C3-4 >2.20 mm, C4-5 >2.60 mm, C5-6 >1.90 mm, C6-7 >1.40 mm (Figure 4) [23].

Anterior head translation

**Figure 5:**
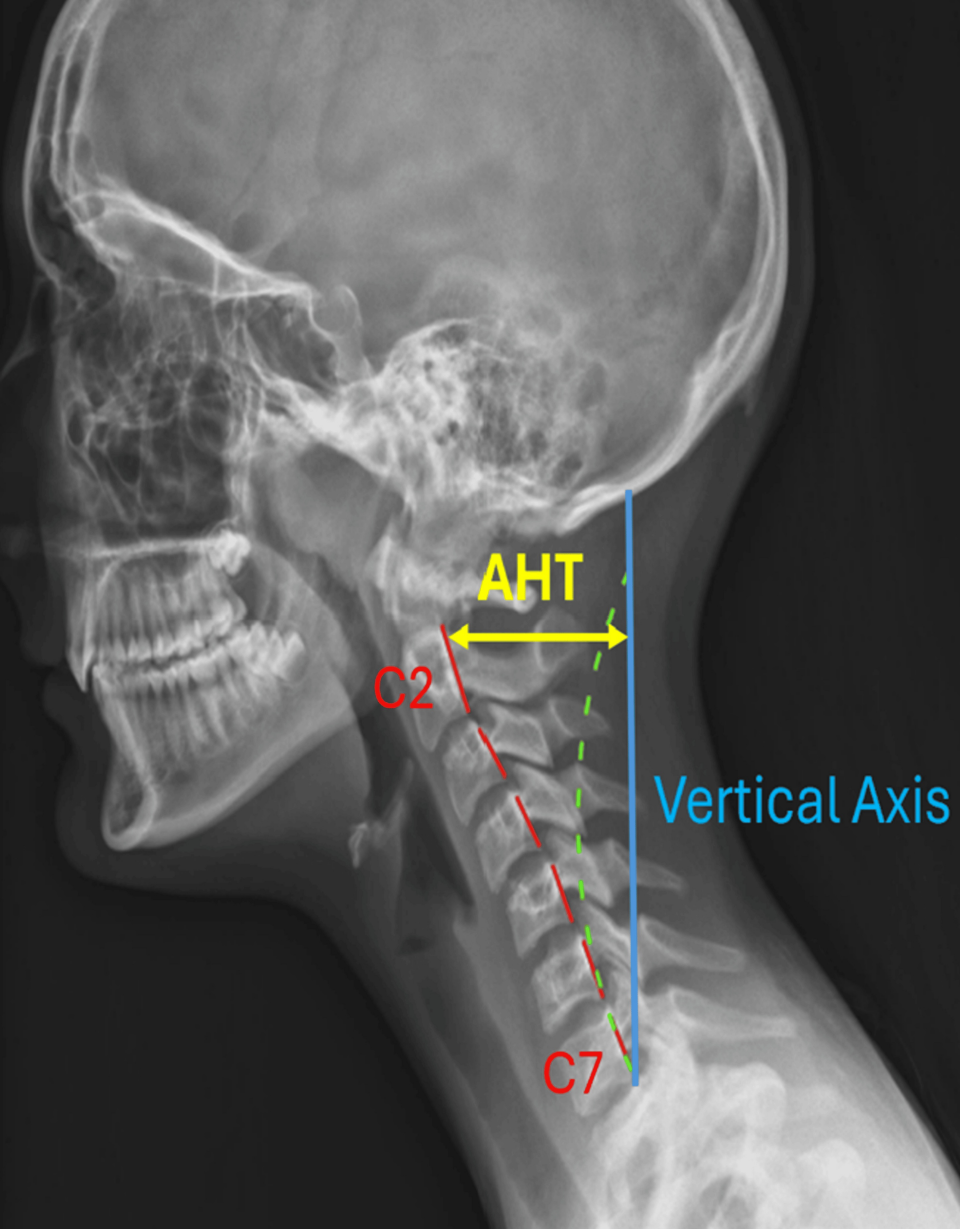
Sagittal view of a lateral neutral cervical radiograph. The green dotted line indicates the estimated normal spinal alignment and the expected path of the posterior longitudinal ligament, while the red line represents the patient's actual spinal alignment and corresponding ligament path. The blue line is a vertical line drawn from the posterior-inferior endplate of C7. The yellow line represents AHT, measured from the posterior-superior endplate of C2 to the vertical axis extending from C7 AHT: anterior head translation

Anterior head translation (AHT) is measured using the thumbs-up right-hand Cartesian coordinate system, where the index finger represents the positive z-axis. The z-axis measurement is taken as a vertical line from the posterior-inferior endplate of C7 to the posterior-superior body of C2 (normal range: 0-20 mm), using PostureRay® software (Figure 5).

## Results

A total of 37 radiographs with scoliosis were included in the final analysis, comprising 11 males (29.7%) and 26 females (70.3%). The average Cobb angle, indicating the severity of spinal curvature, was 35.4° (SD=18.4), with a median of 31.8° and a range from 10.3° to 84.4°. Most radiographs exhibited at least some degree of cervical buckling, with 33 individuals (89.2%) demonstrating one level of buckling and four radiographs (10.8%) presenting with two levels. The mean AHT was 28.3 mm (SD=11.9), with values ranging from 10.6 mm to 54.3 mm (Table 1).

**Table 1:** Descriptive statistics AHT: anterior head translation

Buckling classification, N (%)	
1 buckling	33 (89.2%)
2 buckling	4 (10.8%)
AHT, mean±STD (median, min-max)	28.3±11.9 (25.9, 10.6-54.3)
Abnormal total translation, N (%)	
C2-C3	17 (45.9%)
C3-C4	26 (70.3%)
C4-C5	18 (48.6%)
C5-C6	21 (56.8%)
C6-C7	18 (48.6%)
Abnormal measured angle, N (%)	
C2-C3	8 (21.6%)
C3-C4	3 (8.1%)
C4-C5	1 (2.7%)
C5-C6	1 (2.7%)
C6-C7	1 (2.7%)

Cervical instability: total translation

Abnormal cervical translation, as defined by exceeding normal values, was most frequently observed at the C3-C4 segment (70.3%), followed by C5-C6 (56.8%), C4-C5 and C6-C7 (both 48.6%), and C2-C3 (45.9%). Statistically significant elevation in mean total translation was observed at C3-C4 (mean=2.5 mm, SD=0.6; p=0.0011) and at C5-C6 (mean=2.1 mm, SD=0.7; p=0.0288), suggesting segmental hypermobility relative to normative thresholds. Translation at C2-C3, C4-C5, and C6-C7 did not significantly differ from their respective normal values (p>0.05). The box plot visually supports this, showing elevated interquartile ranges at the C3-C4 and C5-C6 levels.

Cervical instability: measured angulation

Analysis of measured intervertebral angles from C2 through C7 revealed that a small proportion of radiographs exhibited abnormal angulation at any given level. The highest frequency of abnormal measured angles occurred at C2-C3 (21.6%), while all other levels (C3-C7) showed abnormalities in ≤8.1% of cases. However, none of the vertebral segments demonstrated statistically significant deviations in mean angulation when compared to normal values. P-values across all segments were >0.999, indicating no meaningful differences between the observed and normative angular thresholds. This suggests that while translation-based instability was common, angular deformity was much less prevalent in this cohort.

**Figure 6:**
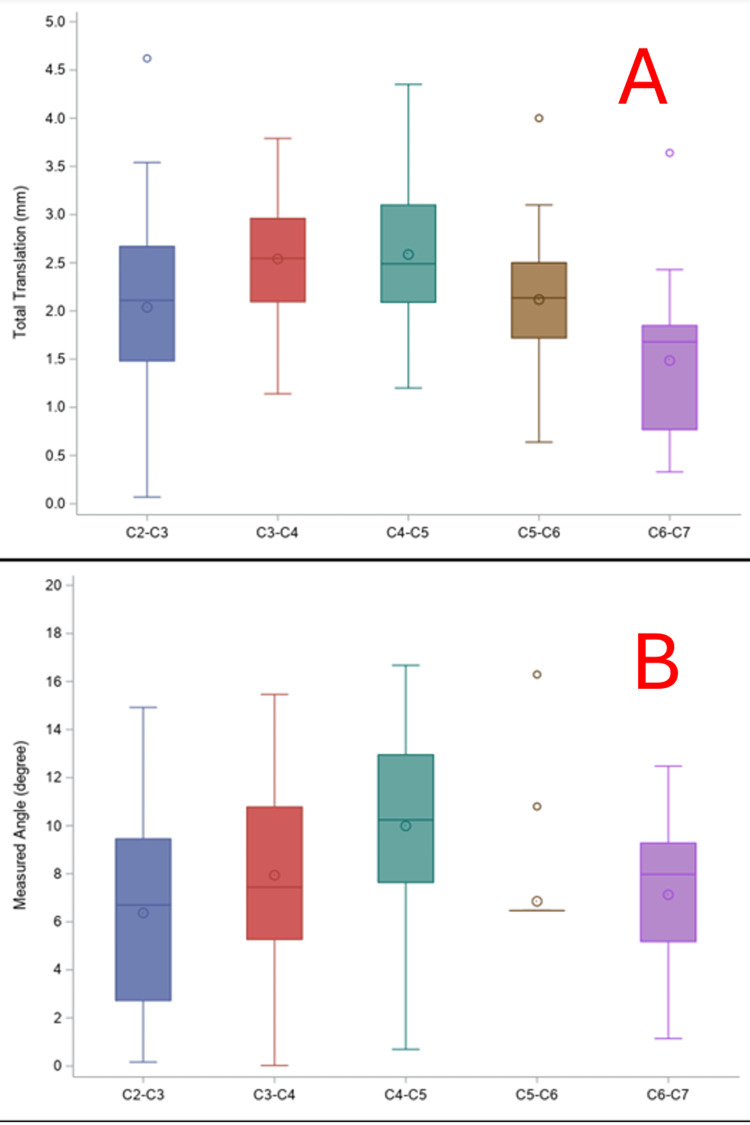
Box plot of total translation (A) and total measured angulation (B) In panel (A), segments C3-C4 and C4-C5 exhibit the highest median translation (~2.5-3 mm). C2-C3 shows a wider range and greater variability, including a low minimum and a high outlier. In panel (B), from C2 through C7, C4-C5 shows the highest median angular motion and range, suggesting it contributes significantly to cervical flexion/extension. C5-C6 appears to have the lowest variability and a low median angle, with several outliers, both high and low, suggesting abnormal kinematic patterns in specific cases.

The overall pattern of our findings indicates that cervical instability in scoliosis radiographs predominantly manifests as increased translational motion, particularly at mid-cervical segments such as C3-C4 and C5-C6. Measured angulation abnormalities were infrequent and did not significantly differ from normal values. This data suggests that translational instability may serve as a more sensitive marker for early biomechanical disturbance disruption in the cervical spine among individuals with scoliosis, particularly in the absence of severe angular deformities (Table 2) (Figure 6).

**Table 2:** Descriptive statistics

Parameters	Mean±STD (median, min-max)	Normal values	P-value
Total translation			
C2-C3	2±1 (2.1, 0.1-4.6)	2.2	0.8366
C3-C4	2.5±0.6 (2.5, 1.1-3.8)	2.2	0.0011
C4-C5	2.6±0.8 (2.5, 1.2-4.4)	2.6	0.5459
C5-C6	2.1±0.7 (2.1, 0.6-4)	1.9	0.0288
C6-C7	1.5±0.7 (1.7, 0.3-3.6)	1.4	0.2695
Measured angle			
C2-C3	6.4±4.2 (6.7, 0.2-14.9)	10	0.9999
C3-C4	7.9±4 (7.4, 0-15.5)	14	0.9999
C4-C5	10±4 (10.2, 0.7-16.7)	16.4	0.9999
C5-C6	6.9±1.7 (6.5, 6.5-16.3)	14.9	0.9999
C6-C7	7.1±2.9 (8, 1.1-12.5)	11.9	0.9999

## Discussion

This study shows that all radiographs in this cohort of AIS patients aged 12 to 20 years with a Risser grade >2 demonstrate cervical instability with cervical buckling. These meet the criteria for the diagnosis-related estimates (DRE) impairment of the cervical region due to ratable loss of motion segment integrity and are at 20%-23% for the whole body [24]. Additionally, this reversal is broken up into different shapes and configurations, comprising 82.2% first-order buckling and 10.8% second-order buckling. The mean instability was measured at C4-C5. These results provide compelling evidence that scoliosis is associated with cervical instability leading to cervical buckling, which disrupts normal spinal biomechanics. Given that many patients exceed surgical intervention thresholds, further studies are needed to explore potential pathogenesis and treatment strategies.

Moustafa et al. (randomized controlled study) demonstrate that a loss of cervical lordosis directly affects the autonomic nervous system (ANS) and sensorimotor control [25]. A systematic review of the literature, Lau et al., demonstrated that proprioceptive deficits occur in all AIS [26]. These studies suggest that abnormal cervical biomechanics are directly linked to neurological dysregulation, limiting the body's ability to effectively regulate the ANS and affecting the neuromuscular system. Collectively, the findings show that abnormal cervical mechanics correlate with abnormal posture and altered biomechanics in regions below the cervical spine. This potentially influences further pathogenesis of the spinal structure. However, Moustafa I, et al. focused only on athletes and performance related to the cervical alignment with no correlation to scoliosis.

Pizones et al. present a prevailing theory regarding the etiopathogenesis of AIS, which posits that dorsally directed shear forces, acting upon a preexisting axial rotation within a posteriorly inclined sagittal profile of the developing thoracic and lumbar spine, initiate rotational instability [27]. This biomechanical model is further modulated by growth-phase disc maturation, collagen quality, and immature proprioceptive feedback. While this framework offers valuable insights, it does not account for the influence of the cervical spine. An oversight that may be significant given its foundational role in governing global spinal biomechanics, central and peripheral neurology, proprioceptive integration, and neuromuscular regulation. The present findings suggest that cervical spine dysfunction, also demonstrating similar factors such as Pizones et al., may serve as an upstream contributor to the cascade of biomechanical and neurological disturbances associated with AIS, warranting greater consideration in future etiological models.

Conservative management of AIS primarily consists of physiotherapy scoliosis-specific exercises (PSSE), bracing, and clinical observation. These interventions are endorsed by the Society on Scoliosis Orthopedic and Rehabilitation Treatment (SOSORT) as evidence-based approaches aimed at preventing curve progression and optimizing spinal function [28]. Surgical intervention is generally advised for skeletally immature individuals with a structural thoracic curve exceeding 45° or for those experiencing continued curve progression despite conservative management [29]. These do not address cervical instability as demonstrated by the association in this AIS case series. Furthermore, the management of cervical instability necessitates careful consideration when assessing readiness for return to sport. As outlined by Huang et al., asymptomatic cervical ligamentous laxity meeting surgical thresholds, for either angulation or translation, is deemed an absolute contraindication to resuming athletic activity [30].

Current treatments for cervical instability include physical therapy, prolotherapy, bracing, corrective chiropractic, and surgical interventions [31,32]. Katz et al. demonstrated that with advanced training, stabilizing cervical instabilities and restoring cervical lordosis has a direct effect on one’s clinical symptoms [33]. Successful conservative treatments for combined cervical instability and AIS include advanced chiropractic approaches, such as those taught by the CLEAR Institute or Chiropractic BioPhysics® [34-37].

This study suggests that AIS patients have a measured cervical instability leading to cervical buckling. The mean instability of C4-C5 consistencies warrants further evaluation as to causation. This pattern is evident in all participants in this study. This buckling changes the mechanics of the cervical spine.

The generalizability of this study is limited by its small sample size and the homogeneity of the cohort, which was drawn from a single geographic region. Larger, multi-center studies are needed to validate these findings and support broader application to the general population. This study lacks a temporal relationship. Furthermore, the study lacks an evaluation of inter- and intra-rater reliability, which is essential to establish the consistency of measurements. Despite these limitations, the consistent and compelling nature of these findings encourages future longitudinal research to further elucidate causative elements and the precise role of cervical mechanics in the pathogenesis and progression of AIS, ultimately guiding more holistic and effective treatment approaches.

The application of these criteria to an adolescent cohort represents a methodological limitation, as pediatric-specific reference values are currently limited, and no association with clinical symptoms was provided. In this study, adult thresholds were utilized pragmatically in the absence of age-appropriate benchmarks. That said, power analysis shows that the sample size in this study is sufficient for the statistical analysis. Additionally, this study does not review any causative elements, and, as such, there is no temporal relationship. Recommendations for future research include: larger population samples over various age groups and monitoring patients over a longer period of time. Following patients from the onset of standing, weight-bearing ages over time may help track the pathogenesis of abnormal spinal alignment, posture, and biomechanical presentations, including instability, buckling, and AHT, to better understand their correlations.

## Conclusions

This study highlights a consistent association between cervical dysfunction and AIS. However, it does not establish a causal relationship. Our sample revealed a 100% prevalence of cervical instability, with all participants exhibiting abnormal translation and fewer (8.1%) showing abnormal angulation. The prevalence of Order 1 (89.2%) and Order 2 (10.8%) buckling further highlights a pervasive biomechanical disturbance in the cervical spine of these patients. This suggests that cervical dysfunction is not merely a secondary finding, but may represent a critical upstream factor influencing the complex spinal biomechanics of AIS. This assessment warrants further evaluation of all AIS diagnoses for dynamic imaging. For healthcare practitioners working with AIS patients, this underscores the vital importance of a comprehensive assessment that includes the cervical spine, recognizing that these comorbidities necessitate simultaneous and integrated treatment strategies.
